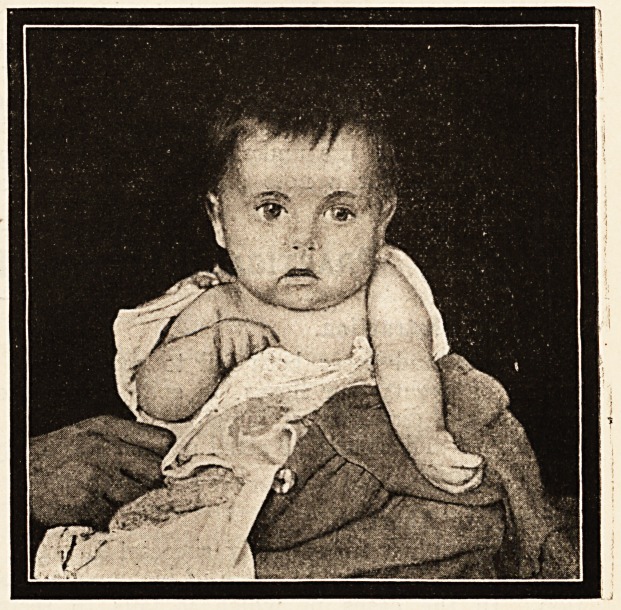# An Epitome of Pediatrics

**Published:** 1910-10-15

**Authors:** 


### AN EPITOME OF PEDIATRICS.*

Goodhart and Still needs no introduction, formal or otherwise, but the new edition of this popular text-book on pediatrics deserves a cordial welcome from the profession. It is one of the best epitomes of that very broad-ranging subject, the diseases of childhood, which exists in the English language. It has always been good, and this new edition has been so considerably revised, added to, and amended, that it may fairly lay claim to rank as one of the foremost works on the subject. Beautifully printed and well illustrated, it is an admirable addition to the practitioner's library, and its conciseness and clearness are added features which should help to extend its popularity and win it more admirers than the already large professional public that now regard it as the text-book on medical diseases of children. During the last decade pediatrics has advanced by leaps and bounds, and is now a recognised speciality, the literature of which is already alarmingly voluminous. The need for an exponent is, therefore, all the more urgent. It is a *sine qua non* that that exponent should be an authority, and that his range should be catholic. Drs. Goodhart and Still's extensive experience gives them the right to pose as such exponents, and the most valuable part of their work is just that personal touch, that egoism which is so attractive in a professional text-book, and which serves to accentuate the individuality of one manual over another, which, so far as its list of contents is concerned, may possess an equally wide range of subjects.

The descriptions in this book are all good, and none err on the side of redundancy; they are solidly instructive, and done in a style which makes their reading attractive to the most wearied reader. As an example of the style of the book and the excellence of its descriptions we may perhaps be allowed to quote the following note on p. 31, to which, by courtesy of Messrs. Churchill, we are permitted to append the illustration that accompanies it:—

Paralysis of the Upper Limb (Erb's Paralysis).—Closely allied in ætiology to sterno-mastoid tumour is the condition known as Erb's paralysis. One of the arms is found at birth, or soon after, to be almost completely paralysed. It hangs flaccid from the shoulder in a very characteristic position; the shoulder appears to be drawn slightly forward, and as the child sits up the arm hangs straight down at the side with the forearm in a position of superpronation, so that the palm of the hand looks backward and outward. The photograph shown here exhibits well the typical position of the arm in Erb's paralysis.

The following case may serve to illustrate it more fully:—

Fred H., aged four months, was brought for weakness of the left arm which had been noticed immediately after birth. Labour had been very protracted, lasting five days; it was a breech presentation; no instruments were used. The infant seems perfectly well in every other way, but the left arm hangs flaccid in a position of superpronation, the palm of the hand looking outward and backward, with the fingers clenched in the palm and the thumb over the fingers. There is no power whatever of flexing the elbow; the muscles of the upper arm are flabby and wasted, especially the deltoid; the bony points about the shoulder are too easily defined; the pectoral muscles are normal; there is some power of voluntary flexion in the fingers. The child remained under our observation for several months; there was then a little increase of movement in the muscles of the forearm, but the upper arm remained unaltered.

"The triceps in these cases is unaffected, the other muscles of the upper arm are paralysed as well as the supinator longus: the supra and infra spinatus may also be paralysed. . . . Rarely the muscles of the forearm are affected much more than those of the upper arm; in this 'lower arm type' there may be no movement of the fingers at all, or flexion may be chiefly affected; the pupil also on the paralysed side may be smaller than on the sound side from injury to the fibres of the sympathetic nerve. Sensation is not affected except in the lower-arm type, when there may be some anæsthesia in the part supplied by the ulnar nerve. Wasting of the affected muscles is very marked as the infant grows older, but, as in muscular atrophy from other causes, it is much less obvious during the first few months of life owing to the amount of subcutaneous fat. The reaction of degeneration is present in the muscles that remain permanently paralysed. The cause of the paralysis is damage to the brachial plexus during delivery, which has usually been difficult, and often instrumental; in many of the cases there has been a breech presentation. The injury is thought to be due to overstretching of the nerve-trunks in most cases, and the usual site of the lesion is in the anterior primary division of the

[1] *The Diseases of Children.* By J. F. Goodhart, M.D., LL.D., F.R.C.P. Ninth edition, edited by G. F. Still, M.D., F.R.C.P. London: J. and A. Churchill, 7 Great Marlborough Street. 1910. Price 15s. net.

fifth cervical nerve. In the rarer lower-arm type the eighth cervical and first dorsal nerves are injured. In a certain number of cases gradual recovery, more or less complete, takes place within a few months, but in the more severe cases, especially if there is reaction of degeneration, the outlook is less hopeful." Then follows an epitome of treatment, in which great stress is rightly laid on operative interference, although it is pointed out that many cases recover without operation.

We apologise to the authors for this long excerpt, but it gives a good idea of the thoroughness of the work which is such a feature of the book, and which makes the volume so useful to the practitioner and student alike. In a text-book like this, compression and condensation must necessarily be carried to extremes, but it is remarkable how excellent the labour of *précis* has been done in this case.

Goodhart and Still is pre-eminently a medical text-book, and it is therefore somewhat unfair to compare it with its popular English rival, Ashby and Wright. We find the surgical sections—and especially those dealing with deformities—rather baldly discussed. Thus, in the article on Lateral Curvature insufficient stress is laid on the fact that in the majority of bad cases a defect of the skeletal system is the underlying cause, although room is found for a note on the rare condition (to which attention has recently been drawn by Fitzwilliams among others) in which an accessory vertebra determines the curve. In the notes on spastic diplegia—which, as regards symptomatology and diagnosis, are quite full—no mention is made of the brilliant work of Spitzy and Codavilla. On the other hand, nothing can be more praiseworthy than the really excellent notes on such more particularly medical lesions as pneumonia, the specific fevers, tuberculosis, functional disorders, heart lesions, and scurvy. In these divisions the book challenges comparison with any other text-book, and appeals to the reader, whether student or practitioner, by its thoroughness. It is pre-eminently a "practical text-book," and the authors give the sum of their personal experiences, not merely the grouping of different methods and diverse opinions. One of the features of the book is the number of illustrative cases, always instructive and well chosen, and reminding us of that half-forgotten, but still useful and absorbingly interesting work, the first edition of Fagge's "Medicine." No higher praise, we imagine, can be given it than to say that it deserves a place in the practitioner's library in line with that monumental (but now, alas! so scarce) text-book which culled its cases largely from the same hospital population which Dr. Goodhart has so effectively exploited—using the verb in its best sense.

## Figures and Tables

**Figure f1:**